# Di-μ-chlorido-bis­{bis­[3,5-dimethyl­pyrazol-1-yl-κ*N* ^2^)methane]iron(II)} bis­[tetra­chloridoferrate(III)]

**DOI:** 10.1107/S1600536808035253

**Published:** 2008-11-08

**Authors:** HongYing Xia, YueLong Liu

**Affiliations:** a College of Chemistry and Chemical Engineering, Jiangxi Science and Technology Normal University, Nanchang 330013, People’s Republic of China

## Abstract

In the title complex, [Fe_2_Cl_2_(C_11_H_16_N_4_)_4_][FeCl_4_]_2_, the asymmetric unit is composed of one and a half units of [Fe(*bdmpm*)_2_Cl]_2_(FeCl_4_)_2_(*bdmpm* = bis(bis(3,5-dimethyl­pyrazol-1-yl)methane). The three independent Fe^II^ atom have a distorted octa­hedral coordination geometry comprising two bridging chloride anions and four N atoms from two bis­(3,5-dimethyl­pyrazol-1-yl)methane ligands. The Fe^III^ atom has a tetra­hedral coordination geometry comprising four chloride anions.

## Related literature

For information on the coordination chemistry of poly-(pyrazol­yl)methane ligands, see: Anderson *et al.* (2000 ▶); Edwards *et al.* (2006 ▶); Higgs *et al.* (1999 ▶); Moubaraki *et al.* (2003 ▶); Pettinari & Pettinari (2005 ▶); Reger *et al.* (2004 ▶). For information on binuclear complexes, see: Moubaraki *et al.* (2003 ▶); Batten *et al.* (2004 ▶); Gu *et al.* (2006 ▶).
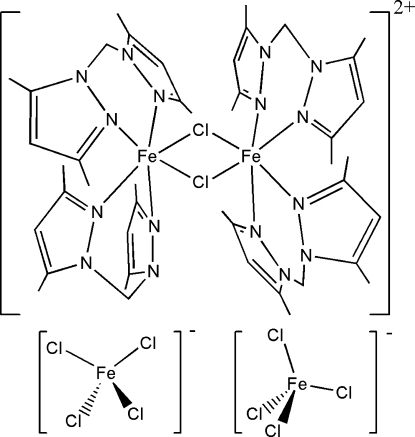


## Experimental

### 

#### Crystal data

[Fe_2_Cl_2_(C_11_H_16_N_4_)_4_][FeCl_4_]_2_
*M* *_r_* = 1395.01
Triclinic, 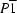

*a* = 14.100 (3) Å
*b* = 18.244 (4) Å
*c* = 19.969 (4) Å
α = 85.58 (3)°
β = 71.23 (3)°
γ = 68.20 (3)°
*V* = 4511 (2) Å^3^
*Z* = 3
Mo *K*α radiation
μ = 1.44 mm^−1^
*T* = 293 (2) K
0.18 × 0.15 × 0.12 mm

#### Data collection

Rigaku Mercury diffractometer
Absorption correction: multi-scan (Jacobson, 1998 ▶) *T*_min_ = 0.782, *T*_max_ = 0.847
44634 measured reflections
16405 independent reflections
10865 reflections with *I* > 2σ(*I*)
*R*_int_ = 0.096

#### Refinement

*R*[*F* ^2^ > 2σ(*F* ^2^)] = 0.093
*wR*(*F* ^2^) = 0.209
*S* = 1.08
16405 reflections
1022 parameters
H-atom parameters constrained
Δρ_max_ = 0.76 e Å^−3^
Δρ_min_ = −0.95 e Å^−3^

### 

Data collection: *CrystalClear* (Rigaku/MSC, 2001 ▶); cell refinement: *CrystalClear*; data reduction: *CrystalStructure* (Rigaku/MSC, 2004 ▶); program(s) used to solve structure: *SHELXS97* (Sheldrick, 2008 ▶); program(s) used to refine structure: *SHELXL97* (Sheldrick, 2008 ▶); molecular graphics: *SHELXTL* (Sheldrick, 2008 ▶); software used to prepare material for publication: *SHELXL97*.

## Supplementary Material

Crystal structure: contains datablocks I, global. DOI: 10.1107/S1600536808035253/br2084sup1.cif

Structure factors: contains datablocks I. DOI: 10.1107/S1600536808035253/br2084Isup2.hkl

Additional supplementary materials:  crystallographic information; 3D view; checkCIF report

## Figures and Tables

**Fig. 1.**
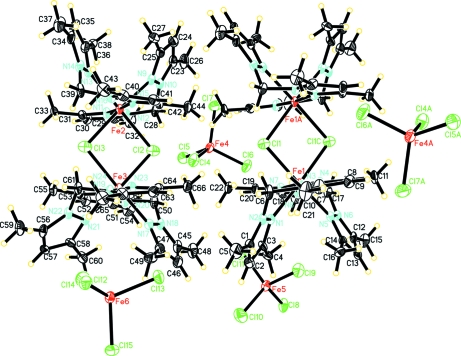
The molecular structure with displacement ellipsoids drawn at the 50% probability level. Atoms labeled with the suffix A are related by the (1 - x, 1 - y, 1 - z) symmetry operator.

## supplementary crystallographic information

### Comment

The various unsubstituted and methyl-substituted poly-(pyrazolyl)methane ligands have become very important in coordination chemistry (Pettinari, *et al.*, 2005; Reger, *et al.*, 2004; Higgs, *et al.*, 1999; Anderson, *et al.*, 2000; Edwards, *et al.*, 2006). But most of them are coordinated with iron to form single nuclear coordination complexes, only few examples are binuclear coordination complexes (Gu, *et al.*, 2006; Batten, *et al.*, 2004; Moubaraki, *et al.*, 2003). In the crystal of the title complex, the asymmetric unit is composed of one and a half units of [Fe(*bdmpm*)_2_Cl]_2_ 2FeCl_4_ (*bdmpm* = bis(bis(3,5-dimethylpyrazol-1-yl)methane).  One of [Fe(*bdmpm*)_2_Cl]_2_ complexes has a crystallographic center of symmetry, the other has an approximate center of symmetry but it is not crystallographic. The reason for this difference is that the bond length and bond angles around Fe(2) and Fe(3) are slightly different. Each binuclear cations with a pair of bridging chloride ligands and the two bmmpm ligands results in six-coordination about each of the metal atoms; the environments of the complex are octahedron geometry (Fig. 1).

### Experimental

The reaction of FeCl_2_.4H_2_O (20 mg, 0.1 mmol) with *bdmpm* (44 mg, 0.22 mmol) in EtOH (15 ml) was carried out at ambient temperature for 20 minutes, the mixture was filtered and the filtrate was then left for crystallization.

### Refinement

In this structrue, the C-H distance was constrained to 0.93 Å for C-H, 0.96 Å for C-H_3_ and 0.97 for C-H_2_. The ratio of the Uiso values 1.2 for H atoms of C-H and C-H_2_, 1.5 for H atoms of C-H_3_.

### Crystal data

**Table:**

[Fe_2_Cl_2_(C_11_H_16_N_4_)_4_][FeCl_4_]_2_	*Z* = 3
*M**_r_* = 1395.01	*F*_000_ = 2142
Triclinic, *P*1	*D*_x_ = 1.541 Mg m^−^^3^
Hall symbol: -P 1	Mo *K*α radiation λ = 0.71073 Å
*a* = 14.100 (3) Å	Cell parameters from 12389 reflections
*b* = 18.244 (4) Å	θ = 3.1–25.4º
*c* = 19.969 (4) Å	µ = 1.44 mm^−^^1^
α = 85.58 (3)º	*T* = 293 (2) K
β = 71.23 (3)º	Prism, yellow
γ = 68.20 (3)º	0.18 × 0.15 × 0.12 mm
*V* = 4511 (2) Å^3^	

### Data collection

**Table:**

Rigaku Mercury diffractometer	16405 independent reflections
Radiation source: fine-focus sealed tube	10865 reflections with *I* > 2σ(*I*)
Monochromator: graphite	*R*_int_ = 0.096
*T* = 293(2) K	θ_max_ = 25.4º
ω scans	θ_min_ = 3.1º
Absorption correction: multi-scan(Jacobson, 1998)	*h* = −14→16
*T*_min_ = 0.782, *T*_max_ = 0.847	*k* = −21→21
44634 measured reflections	*l* = −22→24

### Refinement

**Table:**

Refinement on *F*^2^	Secondary atom site location: difference Fourier map
Least-squares matrix: full	Hydrogen site location: inferred from neighbouring sites
*R*[*F*^2^ > 2σ(*F*^2^)] = 0.093	H-atom parameters constrained
*wR*(*F*^2^) = 0.209	*w* = 1/[σ^2^(*F*_o_^2^) + (0.0667*P*)^2^ + 17.766*P*] where *P* = (*F*_o_^2^ + 2*F*_c_^2^)/3
*S* = 1.08	(Δ/σ)_max_ = 0.001
16405 reflections	Δρ_max_ = 0.76 e Å^−^^3^
1022 parameters	Δρ_min_ = −0.95 e Å^−^^3^
Primary atom site location: structure-invariant direct methods	Extinction correction: none

### Special details

**Table:**

Geometry. All e.s.d.'s (except the e.s.d. in the dihedral angle between two l.s. planes) are estimated using the full covariance matrix. The cell e.s.d.'s are taken into account individually in the estimation of e.s.d.'s in distances, angles and torsion angles; correlations between e.s.d.'s in cell parameters are only used when they are defined by crystal symmetry. An approximate (isotropic) treatment of cell e.s.d.'s is used for estimating e.s.d.'s involving l.s. planes.
Refinement. Refinement of *F*^2^ against ALL reflections. The weighted *R*-factor *wR* and goodness of fit *S* are based on *F*^2^, conventional *R*-factors *R* are based on *F*, with *F* set to zero for negative *F*^2^. The threshold expression of *F*^2^ > σ(*F*^2^) is used only for calculating *R*-factors(gt) *etc*. and is not relevant to the choice of reflections for refinement. *R*-factors based on *F*^2^ are statistically about twice as large as those based on *F*, and *R*- factors based on ALL data will be even larger.

### Fractional atomic coordinates and isotropic or equivalent isotropic displacement parameters (Å^2^)

**Table:**

	*x*	*y*	*z*	*U*_iso_*/*U*_eq_	
Fe1	0.62332 (8)	0.51099 (6)	0.50914 (5)	0.0247 (2)	
Fe2	0.74539 (9)	0.16729 (6)	0.16291 (5)	0.0257 (3)	
Fe3	0.99665 (9)	0.17009 (6)	0.18208 (5)	0.0282 (3)	
Fe4	0.63770 (10)	0.63582 (7)	0.06660 (6)	0.0422 (3)	
Fe5	0.99828 (10)	0.69688 (7)	0.26964 (6)	0.0370 (3)	
Fe6	1.32478 (10)	−0.03970 (7)	0.40487 (6)	0.0354 (3)	
Cl1	0.57354 (17)	0.41434 (12)	0.46044 (11)	0.0410 (5)	
Cl2	0.80653 (18)	0.25535 (13)	0.21295 (12)	0.0477 (5)	
Cl3	0.93390 (19)	0.08437 (14)	0.13036 (12)	0.0514 (6)	
Cl4	0.7281 (2)	0.52573 (14)	0.10930 (12)	0.0573 (6)	
Cl5	0.7239 (2)	0.64291 (16)	−0.04497 (12)	0.0610 (7)	
Cl6	0.6167 (2)	0.73748 (16)	0.12761 (16)	0.0754 (8)	
Cl7	0.4792 (2)	0.63502 (19)	0.07611 (13)	0.0707 (8)	
Cl8	1.0250 (2)	0.80953 (13)	0.24793 (12)	0.0587 (7)	
Cl9	0.8800 (2)	0.70590 (15)	0.37419 (12)	0.0602 (7)	
Cl10	1.1519 (2)	0.60330 (17)	0.26359 (17)	0.0781 (8)	
Cl11	0.9354 (3)	0.67382 (17)	0.19135 (15)	0.0774 (9)	
Cl12	1.31887 (19)	−0.15580 (13)	0.44282 (12)	0.0499 (6)	
Cl13	1.18150 (19)	0.05457 (13)	0.46825 (13)	0.0533 (6)	
Cl14	1.3308 (2)	−0.03182 (17)	0.29382 (12)	0.0652 (7)	
Cl15	1.46816 (19)	−0.03050 (15)	0.41644 (14)	0.0567 (6)	
N1	0.7937 (5)	0.4304 (3)	0.4892 (3)	0.0261 (14)	
N2	0.8140 (5)	0.3594 (3)	0.5215 (3)	0.0293 (14)	
N3	0.6535 (5)	0.3821 (3)	0.6202 (3)	0.0312 (15)	
N4	0.5976 (5)	0.4607 (3)	0.6133 (3)	0.0275 (14)	
N5	0.6583 (5)	0.6072 (3)	0.5454 (3)	0.0268 (14)	
N6	0.6305 (5)	0.6792 (3)	0.5136 (3)	0.0276 (14)	
N7	0.6583 (5)	0.5625 (3)	0.4070 (3)	0.0260 (13)	
N8	0.6266 (5)	0.6434 (3)	0.4021 (3)	0.0284 (14)	
N9	0.5806 (5)	0.2555 (3)	0.1851 (3)	0.0302 (15)	
N10	0.5666 (5)	0.3242 (3)	0.1493 (3)	0.0300 (14)	
N11	0.7731 (5)	0.2163 (3)	0.0589 (3)	0.0285 (14)	
N12	0.7309 (5)	0.2976 (3)	0.0525 (3)	0.0289 (14)	
N13	0.6936 (5)	0.0789 (4)	0.1283 (3)	0.0326 (15)	
N14	0.7025 (5)	0.0118 (4)	0.1674 (3)	0.0352 (16)	
N15	0.7092 (5)	0.1155 (3)	0.2663 (3)	0.0262 (14)	
N16	0.7415 (5)	0.0349 (3)	0.2707 (3)	0.0314 (15)	
N17	1.0463 (5)	0.2590 (4)	0.2160 (3)	0.0335 (15)	
N18	1.0214 (5)	0.3315 (3)	0.1877 (3)	0.0354 (16)	
N19	1.0364 (5)	0.2189 (3)	0.0794 (3)	0.0331 (15)	
N20	1.0169 (6)	0.2978 (4)	0.0749 (3)	0.0395 (17)	
N21	1.1602 (5)	0.0799 (3)	0.1588 (3)	0.0306 (14)	
N22	1.1697 (5)	0.0043 (3)	0.1781 (3)	0.0304 (14)	
N23	0.9701 (5)	0.1183 (3)	0.2847 (3)	0.0286 (14)	
N24	1.0253 (5)	0.0387 (3)	0.2891 (3)	0.0310 (15)	
C1	0.9188 (7)	0.3241 (4)	0.5144 (4)	0.037 (2)	
C2	0.9694 (7)	0.3718 (5)	0.4769 (4)	0.041 (2)	
H2A	1.0426	0.3623	0.4636	0.049*	
C3	0.8891 (6)	0.4383 (4)	0.4623 (4)	0.0307 (17)	
C4	0.9045 (6)	0.5082 (5)	0.4232 (4)	0.0370 (19)	
H4A	0.9301	0.4958	0.3731	0.055*	
H4B	0.9562	0.5216	0.4361	0.055*	
H4C	0.8371	0.5522	0.4350	0.055*	
C5	0.9646 (8)	0.2436 (5)	0.5413 (5)	0.059 (3)	
H5A	0.9369	0.2460	0.5920	0.088*	
H5B	1.0417	0.2265	0.5265	0.088*	
H5C	0.9444	0.2068	0.5224	0.088*	
C6	0.7271 (7)	0.3339 (4)	0.5571 (4)	0.037 (2)	
H6A	0.7561	0.2796	0.5699	0.045*	
H6B	0.6876	0.3351	0.5249	0.045*	
C7	0.6286 (7)	0.3620 (4)	0.6881 (4)	0.039 (2)	
C8	0.5552 (7)	0.4292 (5)	0.7274 (4)	0.039 (2)	
H8A	0.5224	0.4336	0.7762	0.047*	
C9	0.5391 (6)	0.4899 (4)	0.6802 (4)	0.0303 (17)	
C10	0.6762 (9)	0.2811 (5)	0.7122 (5)	0.060 (3)	
H10A	0.6638	0.2438	0.6880	0.090*	
H10B	0.6432	0.2800	0.7623	0.090*	
H10C	0.7523	0.2673	0.7019	0.090*	
C11	0.4729 (7)	0.5744 (5)	0.6969 (4)	0.041 (2)	
H11A	0.5104	0.5992	0.7142	0.062*	
H11B	0.4057	0.5799	0.7326	0.062*	
H11C	0.4595	0.5992	0.6550	0.062*	
C12	0.6665 (6)	0.7291 (4)	0.5355 (4)	0.0317 (18)	
C13	0.7196 (6)	0.6896 (4)	0.5815 (4)	0.0314 (18)	
H13A	0.7532	0.7087	0.6053	0.038*	
C14	0.7132 (6)	0.6144 (4)	0.5854 (4)	0.0285 (17)	
C15	0.6445 (8)	0.8130 (4)	0.5129 (5)	0.048 (2)	
H15A	0.6693	0.8131	0.4621	0.072*	
H15B	0.6818	0.8364	0.5320	0.072*	
H15C	0.5686	0.8429	0.5302	0.072*	
C16	0.7610 (7)	0.5477 (5)	0.6277 (4)	0.0364 (19)	
H16A	0.7177	0.5576	0.6766	0.055*	
H16B	0.8328	0.5434	0.6231	0.055*	
H16C	0.7632	0.4992	0.6105	0.055*	
C17	0.5678 (6)	0.6935 (4)	0.4663 (4)	0.0299 (17)	
H17A	0.5479	0.7485	0.4540	0.036*	
H17B	0.5021	0.6839	0.4901	0.036*	
C18	0.6542 (7)	0.6650 (5)	0.3351 (4)	0.0335 (18)	
C19	0.7084 (6)	0.5961 (4)	0.2934 (4)	0.0325 (18)	
H19A	0.7378	0.5916	0.2443	0.039*	
C20	0.7103 (6)	0.5347 (4)	0.3391 (4)	0.0302 (17)	
C21	0.6302 (8)	0.7493 (5)	0.3150 (5)	0.051 (2)	
H21A	0.5905	0.7837	0.3567	0.077*	
H21B	0.5881	0.7609	0.2833	0.077*	
H21C	0.6964	0.7574	0.2918	0.077*	
C22	0.7658 (7)	0.4472 (4)	0.3209 (4)	0.038 (2)	
H22A	0.7533	0.4186	0.3632	0.057*	
H22B	0.8418	0.4347	0.2994	0.057*	
H22C	0.7377	0.4325	0.2883	0.057*	
C23	0.4612 (7)	0.3631 (5)	0.1552 (4)	0.040 (2)	
C24	0.4075 (7)	0.3191 (5)	0.1935 (4)	0.044 (2)	
H24A	0.3339	0.3304	0.2053	0.053*	
C25	0.4811 (7)	0.2538 (5)	0.2124 (4)	0.0358 (19)	
C26	0.4232 (8)	0.4413 (5)	0.1214 (5)	0.057 (3)	
H26A	0.3463	0.4602	0.1325	0.086*	
H26B	0.4563	0.4341	0.0710	0.086*	
H26C	0.4429	0.4790	0.1392	0.086*	
C27	0.4616 (7)	0.1858 (5)	0.2536 (5)	0.047 (2)	
H27A	0.4306	0.1612	0.2302	0.070*	
H27B	0.4131	0.2046	0.3004	0.070*	
H27C	0.5286	0.1479	0.2568	0.070*	
C28	0.6579 (7)	0.3472 (4)	0.1162 (4)	0.0356 (19)	
H28A	0.6966	0.3430	0.1495	0.043*	
H28B	0.6328	0.4020	0.1037	0.043*	
C29	0.7608 (7)	0.3186 (5)	−0.0149 (4)	0.0341 (18)	
C30	0.8243 (7)	0.2482 (5)	−0.0549 (4)	0.038 (2)	
H30A	0.8582	0.2428	−0.1036	0.045*	
C31	0.8275 (6)	0.1868 (4)	−0.0076 (4)	0.0283 (17)	
C32	0.7313 (8)	0.4014 (5)	−0.0373 (4)	0.049 (2)	
H32A	0.6544	0.4259	−0.0250	0.073*	
H32B	0.7631	0.4017	−0.0877	0.073*	
H32C	0.7574	0.4301	−0.0139	0.073*	
C33	0.8799 (6)	0.1007 (5)	−0.0257 (4)	0.0366 (19)	
H33A	0.8965	0.0734	0.0146	0.055*	
H33B	0.9450	0.0905	−0.0647	0.055*	
H33C	0.8320	0.0826	−0.0385	0.055*	
C34	0.6352 (7)	−0.0221 (5)	0.1630 (5)	0.044 (2)	
C35	0.5815 (8)	0.0232 (5)	0.1197 (4)	0.046 (2)	
H35A	0.5287	0.0148	0.1065	0.055*	
C36	0.6196 (7)	0.0847 (5)	0.0986 (4)	0.042 (2)	
C37	0.6298 (9)	−0.0956 (6)	0.2013 (5)	0.065 (3)	
H37A	0.6991	−0.1375	0.1858	0.097*	
H37B	0.5775	−0.1110	0.1912	0.097*	
H37C	0.6092	−0.0852	0.2514	0.097*	
C38	0.5838 (7)	0.1494 (5)	0.0528 (4)	0.042 (2)	
H38A	0.5110	0.1832	0.0766	0.063*	
H38B	0.5874	0.1277	0.0094	0.063*	
H38C	0.6297	0.1794	0.0427	0.063*	
C39	0.7792 (7)	−0.0124 (4)	0.2052 (4)	0.037 (2)	
H39A	0.8461	−0.0080	0.1752	0.045*	
H39B	0.7938	−0.0674	0.2162	0.045*	
C40	0.7230 (6)	0.0123 (5)	0.3382 (4)	0.0361 (19)	
C41	0.6764 (6)	0.0800 (5)	0.3797 (4)	0.0340 (18)	
H41A	0.6546	0.0839	0.4289	0.041*	
C42	0.6674 (6)	0.1431 (4)	0.3340 (4)	0.0289 (17)	
C43	0.7498 (7)	−0.0714 (5)	0.3587 (5)	0.046 (2)	
H43A	0.7126	−0.0956	0.3404	0.069*	
H43B	0.7280	−0.0731	0.4094	0.069*	
H43C	0.8260	−0.0996	0.3395	0.069*	
C44	0.6167 (7)	0.2287 (4)	0.3534 (4)	0.039 (2)	
H44A	0.6481	0.2570	0.3159	0.059*	
H44B	0.6283	0.2385	0.3961	0.059*	
H44C	0.5408	0.2461	0.3608	0.059*	
C45	1.0586 (7)	0.3788 (5)	0.2121 (4)	0.043 (2)	
C46	1.1065 (8)	0.3363 (6)	0.2584 (5)	0.055 (3)	
H46A	1.1384	0.3536	0.2848	0.065*	
C47	1.1000 (7)	0.2623 (5)	0.2598 (4)	0.038 (2)	
C48	1.0394 (9)	0.4629 (5)	0.1916 (5)	0.062 (3)	
H48A	1.0713	0.4639	0.1414	0.093*	
H48B	1.0712	0.4859	0.2158	0.093*	
H48C	0.9633	0.4925	0.2046	0.093*	
C49	1.1465 (7)	0.1917 (5)	0.2990 (5)	0.048 (2)	
H49A	1.1056	0.2003	0.3484	0.073*	
H49B	1.2200	0.1838	0.2931	0.073*	
H49C	1.1438	0.1457	0.2806	0.073*	
C50	0.9607 (8)	0.3492 (4)	0.1381 (4)	0.044 (2)	
H50A	0.8919	0.3437	0.1611	0.052*	
H50B	0.9467	0.4036	0.1250	0.052*	
C51	1.0533 (7)	0.3169 (5)	0.0074 (4)	0.0365 (19)	
C52	1.0996 (7)	0.2488 (5)	−0.0330 (4)	0.039 (2)	
H52A	1.1317	0.2436	−0.0819	0.047*	
C53	1.0903 (6)	0.1880 (4)	0.0121 (4)	0.0314 (17)	
C54	1.0374 (9)	0.4006 (5)	−0.0139 (5)	0.055 (3)	
H54A	0.9618	0.4311	−0.0029	0.082*	
H54B	1.0723	0.4007	−0.0638	0.082*	
H54C	1.0680	0.4233	0.0115	0.082*	
C55	1.1312 (7)	0.1023 (4)	−0.0065 (4)	0.043 (2)	
H55A	1.1041	0.0754	0.0340	0.064*	
H55B	1.2085	0.0817	−0.0212	0.064*	
H55C	1.1076	0.0943	−0.0445	0.064*	
C56	1.2710 (7)	−0.0487 (4)	0.1468 (4)	0.0361 (19)	
C57	1.3278 (6)	−0.0050 (4)	0.1076 (4)	0.0355 (19)	
H57A	1.3997	−0.0245	0.0795	0.043*	
C58	1.2595 (6)	0.0729 (5)	0.1173 (4)	0.0331 (18)	
C59	1.3022 (7)	−0.1345 (4)	0.1588 (4)	0.044 (2)	
H59A	1.2889	−0.1426	0.2085	0.067*	
H59B	1.3774	−0.1612	0.1340	0.067*	
H59C	1.2605	−0.1553	0.1417	0.067*	
C60	1.2860 (7)	0.1434 (5)	0.0887 (5)	0.045 (2)	
H60A	1.2216	0.1899	0.1013	0.068*	
H60B	1.3164	0.1369	0.0381	0.068*	
H60C	1.3371	0.1487	0.1084	0.068*	
C61	1.0760 (6)	−0.0108 (4)	0.2252 (4)	0.0312 (18)	
H61A	1.0241	−0.0018	0.2004	0.037*	
H61B	1.0983	−0.0657	0.2374	0.037*	
C62	1.0109 (6)	0.0187 (5)	0.3568 (4)	0.0337 (18)	
C63	0.9459 (7)	0.0861 (5)	0.3978 (4)	0.0380 (19)	
H63A	0.9216	0.0904	0.4470	0.046*	
C64	0.9228 (6)	0.1473 (5)	0.3524 (4)	0.0314 (18)	
C65	1.0595 (7)	−0.0641 (5)	0.3771 (5)	0.047 (2)	
H65A	1.0305	−0.0973	0.3616	0.071*	
H65B	1.0431	−0.0652	0.4276	0.071*	
H65C	1.1362	−0.0832	0.3551	0.071*	
C66	0.8590 (7)	0.2325 (5)	0.3715 (4)	0.042 (2)	
H66A	0.9027	0.2567	0.3822	0.062*	
H66B	0.7972	0.2381	0.4122	0.062*	
H66C	0.8359	0.2577	0.3325	0.062*	

### Atomic displacement parameters (Å^2^)

**Table:**

	*U*^11^	*U*^22^	*U*^33^	*U*^12^	*U*^13^	*U*^23^
Fe1	0.0325 (6)	0.0166 (5)	0.0198 (5)	−0.0057 (5)	−0.0057 (4)	0.0023 (4)
Fe2	0.0330 (6)	0.0174 (5)	0.0225 (5)	−0.0082 (5)	−0.0053 (5)	0.0036 (4)
Fe3	0.0371 (7)	0.0172 (5)	0.0250 (6)	−0.0074 (5)	−0.0066 (5)	0.0036 (4)
Fe4	0.0518 (8)	0.0402 (7)	0.0364 (7)	−0.0215 (6)	−0.0093 (6)	−0.0052 (5)
Fe5	0.0518 (8)	0.0269 (6)	0.0343 (6)	−0.0156 (6)	−0.0157 (6)	0.0054 (5)
Fe6	0.0405 (7)	0.0314 (7)	0.0330 (6)	−0.0088 (5)	−0.0145 (5)	−0.0018 (5)
Cl1	0.0454 (12)	0.0432 (12)	0.0421 (11)	−0.0224 (10)	−0.0191 (10)	0.0132 (9)
Cl2	0.0463 (13)	0.0519 (14)	0.0517 (13)	−0.0225 (11)	−0.0226 (11)	0.0182 (10)
Cl3	0.0502 (14)	0.0557 (15)	0.0560 (14)	−0.0252 (12)	−0.0239 (11)	0.0191 (11)
Cl4	0.0776 (18)	0.0479 (14)	0.0466 (13)	−0.0239 (13)	−0.0195 (12)	0.0035 (10)
Cl5	0.0685 (17)	0.0799 (19)	0.0388 (13)	−0.0368 (15)	−0.0125 (12)	0.0089 (12)
Cl6	0.083 (2)	0.0571 (17)	0.090 (2)	−0.0209 (15)	−0.0305 (16)	−0.0304 (15)
Cl7	0.0692 (18)	0.113 (2)	0.0486 (14)	−0.0567 (18)	−0.0141 (13)	−0.0005 (14)
Cl8	0.0803 (18)	0.0387 (13)	0.0496 (14)	−0.0338 (13)	0.0047 (12)	−0.0043 (10)
Cl9	0.0774 (18)	0.0578 (16)	0.0436 (13)	−0.0367 (14)	−0.0048 (12)	0.0104 (11)
Cl10	0.0682 (19)	0.0559 (17)	0.094 (2)	0.0004 (14)	−0.0334 (16)	0.0099 (15)
Cl11	0.131 (3)	0.0736 (19)	0.0648 (17)	−0.0565 (19)	−0.0611 (18)	0.0231 (14)
Cl12	0.0556 (14)	0.0343 (12)	0.0626 (14)	−0.0162 (11)	−0.0227 (12)	0.0035 (10)
Cl13	0.0453 (13)	0.0405 (13)	0.0596 (14)	−0.0010 (11)	−0.0122 (11)	−0.0106 (10)
Cl14	0.0726 (18)	0.086 (2)	0.0361 (12)	−0.0237 (15)	−0.0222 (12)	0.0028 (12)
Cl15	0.0464 (14)	0.0592 (16)	0.0727 (17)	−0.0207 (12)	−0.0265 (12)	−0.0040 (12)
N1	0.039 (4)	0.012 (3)	0.026 (3)	−0.007 (3)	−0.012 (3)	0.004 (2)
N2	0.039 (4)	0.013 (3)	0.029 (3)	−0.004 (3)	−0.009 (3)	0.001 (2)
N3	0.046 (4)	0.015 (3)	0.019 (3)	−0.001 (3)	−0.006 (3)	0.003 (2)
N4	0.040 (4)	0.020 (3)	0.020 (3)	−0.009 (3)	−0.007 (3)	0.001 (2)
N5	0.030 (3)	0.016 (3)	0.028 (3)	−0.005 (3)	−0.007 (3)	0.004 (2)
N6	0.030 (4)	0.017 (3)	0.030 (3)	−0.005 (3)	−0.005 (3)	0.001 (2)
N7	0.030 (3)	0.016 (3)	0.027 (3)	−0.009 (3)	−0.003 (3)	0.005 (2)
N8	0.043 (4)	0.021 (3)	0.019 (3)	−0.012 (3)	−0.007 (3)	0.005 (2)
N9	0.045 (4)	0.023 (3)	0.020 (3)	−0.011 (3)	−0.009 (3)	0.001 (2)
N10	0.042 (4)	0.019 (3)	0.025 (3)	−0.009 (3)	−0.008 (3)	0.002 (2)
N11	0.039 (4)	0.017 (3)	0.023 (3)	−0.007 (3)	−0.006 (3)	0.006 (2)
N12	0.042 (4)	0.020 (3)	0.022 (3)	−0.007 (3)	−0.012 (3)	0.003 (2)
N13	0.042 (4)	0.025 (4)	0.029 (3)	−0.015 (3)	−0.005 (3)	0.001 (3)
N14	0.046 (4)	0.019 (3)	0.036 (4)	−0.012 (3)	−0.007 (3)	0.002 (3)
N15	0.040 (4)	0.014 (3)	0.023 (3)	−0.011 (3)	−0.006 (3)	0.001 (2)
N16	0.041 (4)	0.024 (3)	0.023 (3)	−0.012 (3)	−0.002 (3)	0.006 (3)
N17	0.038 (4)	0.024 (4)	0.037 (4)	−0.015 (3)	−0.007 (3)	0.005 (3)
N18	0.042 (4)	0.018 (3)	0.039 (4)	−0.009 (3)	−0.005 (3)	−0.004 (3)
N19	0.050 (4)	0.013 (3)	0.029 (3)	−0.009 (3)	−0.006 (3)	0.006 (2)
N20	0.056 (5)	0.021 (3)	0.031 (4)	−0.013 (3)	−0.002 (3)	0.005 (3)
N21	0.034 (4)	0.017 (3)	0.039 (4)	−0.006 (3)	−0.013 (3)	0.002 (3)
N22	0.038 (4)	0.020 (3)	0.026 (3)	−0.006 (3)	−0.006 (3)	0.004 (3)
N23	0.036 (4)	0.019 (3)	0.028 (3)	−0.005 (3)	−0.012 (3)	0.006 (2)
N24	0.044 (4)	0.017 (3)	0.027 (3)	−0.009 (3)	−0.008 (3)	0.002 (2)
C1	0.039 (5)	0.022 (4)	0.042 (5)	0.006 (4)	−0.022 (4)	−0.004 (3)
C2	0.036 (5)	0.034 (5)	0.049 (5)	−0.006 (4)	−0.014 (4)	−0.005 (4)
C3	0.021 (4)	0.021 (4)	0.038 (4)	−0.002 (3)	−0.001 (3)	0.000 (3)
C4	0.026 (4)	0.040 (5)	0.042 (5)	−0.013 (4)	−0.005 (4)	0.003 (4)
C5	0.068 (7)	0.031 (5)	0.068 (6)	0.007 (5)	−0.040 (6)	0.005 (4)
C6	0.059 (6)	0.013 (4)	0.026 (4)	−0.006 (4)	−0.003 (4)	0.002 (3)
C7	0.059 (6)	0.024 (4)	0.029 (4)	−0.013 (4)	−0.011 (4)	0.012 (3)
C8	0.054 (6)	0.043 (5)	0.024 (4)	−0.025 (5)	−0.010 (4)	0.008 (4)
C9	0.026 (4)	0.035 (5)	0.024 (4)	−0.011 (4)	−0.002 (3)	0.001 (3)
C10	0.106 (9)	0.028 (5)	0.036 (5)	−0.014 (5)	−0.027 (5)	0.021 (4)
C11	0.042 (5)	0.033 (5)	0.031 (4)	0.005 (4)	−0.005 (4)	−0.015 (4)
C12	0.040 (5)	0.015 (4)	0.031 (4)	−0.009 (3)	0.000 (4)	−0.006 (3)
C13	0.028 (4)	0.020 (4)	0.039 (4)	−0.006 (3)	−0.003 (4)	−0.008 (3)
C14	0.027 (4)	0.024 (4)	0.026 (4)	0.000 (3)	−0.008 (3)	−0.001 (3)
C15	0.073 (7)	0.020 (4)	0.049 (5)	−0.022 (4)	−0.011 (5)	0.006 (4)
C16	0.041 (5)	0.035 (5)	0.040 (5)	−0.015 (4)	−0.022 (4)	0.003 (4)
C17	0.045 (5)	0.019 (4)	0.025 (4)	−0.011 (4)	−0.012 (4)	0.004 (3)
C18	0.046 (5)	0.032 (4)	0.030 (4)	−0.021 (4)	−0.016 (4)	0.012 (3)
C19	0.042 (5)	0.033 (4)	0.020 (4)	−0.018 (4)	0.000 (3)	−0.004 (3)
C20	0.036 (4)	0.024 (4)	0.028 (4)	−0.013 (4)	−0.002 (3)	−0.007 (3)
C21	0.078 (7)	0.039 (5)	0.040 (5)	−0.027 (5)	−0.021 (5)	0.018 (4)
C22	0.050 (5)	0.035 (5)	0.026 (4)	−0.020 (4)	−0.001 (4)	−0.007 (3)
C23	0.045 (5)	0.030 (5)	0.035 (5)	0.005 (4)	−0.021 (4)	−0.005 (4)
C24	0.040 (5)	0.047 (6)	0.042 (5)	−0.009 (5)	−0.014 (4)	−0.004 (4)
C25	0.035 (5)	0.037 (5)	0.032 (4)	−0.009 (4)	−0.009 (4)	−0.007 (3)
C26	0.069 (7)	0.039 (5)	0.050 (6)	0.007 (5)	−0.029 (5)	−0.008 (4)
C27	0.036 (5)	0.052 (6)	0.050 (5)	−0.019 (4)	−0.004 (4)	−0.006 (4)
C28	0.059 (6)	0.018 (4)	0.024 (4)	−0.009 (4)	−0.013 (4)	0.001 (3)
C29	0.048 (5)	0.033 (5)	0.027 (4)	−0.020 (4)	−0.016 (4)	0.014 (3)
C30	0.049 (5)	0.040 (5)	0.023 (4)	−0.021 (4)	−0.005 (4)	0.003 (3)
C31	0.028 (4)	0.027 (4)	0.026 (4)	−0.008 (3)	−0.006 (3)	0.002 (3)
C32	0.075 (7)	0.038 (5)	0.037 (5)	−0.025 (5)	−0.020 (5)	0.012 (4)
C33	0.035 (5)	0.037 (5)	0.025 (4)	−0.004 (4)	−0.002 (3)	−0.007 (3)
C34	0.056 (6)	0.024 (4)	0.043 (5)	−0.021 (4)	0.007 (4)	−0.011 (4)
C35	0.063 (6)	0.051 (6)	0.036 (5)	−0.039 (5)	−0.007 (4)	−0.008 (4)
C36	0.047 (5)	0.036 (5)	0.040 (5)	−0.008 (4)	−0.014 (4)	−0.009 (4)
C37	0.087 (8)	0.046 (6)	0.055 (6)	−0.040 (6)	0.008 (6)	−0.003 (5)
C38	0.042 (5)	0.045 (5)	0.044 (5)	−0.017 (4)	−0.017 (4)	−0.009 (4)
C39	0.050 (5)	0.012 (4)	0.028 (4)	−0.004 (4)	0.005 (4)	0.004 (3)
C40	0.037 (5)	0.038 (5)	0.032 (4)	−0.014 (4)	−0.011 (4)	0.014 (4)
C41	0.033 (4)	0.049 (5)	0.019 (4)	−0.017 (4)	−0.007 (3)	0.010 (3)
C42	0.030 (4)	0.023 (4)	0.026 (4)	−0.005 (3)	−0.005 (3)	0.000 (3)
C43	0.046 (5)	0.037 (5)	0.048 (5)	−0.010 (4)	−0.016 (4)	0.020 (4)
C44	0.042 (5)	0.031 (5)	0.036 (4)	−0.009 (4)	−0.004 (4)	−0.011 (3)
C45	0.057 (6)	0.032 (5)	0.033 (5)	−0.024 (4)	0.006 (4)	−0.004 (4)
C46	0.053 (6)	0.057 (6)	0.051 (6)	−0.032 (5)	0.004 (5)	−0.019 (5)
C47	0.034 (5)	0.033 (5)	0.043 (5)	−0.014 (4)	−0.004 (4)	−0.003 (4)
C48	0.079 (8)	0.030 (5)	0.064 (7)	−0.028 (5)	0.003 (6)	−0.003 (4)
C49	0.046 (6)	0.061 (6)	0.048 (5)	−0.020 (5)	−0.026 (4)	0.010 (4)
C50	0.062 (6)	0.016 (4)	0.037 (5)	−0.003 (4)	−0.007 (4)	0.002 (3)
C51	0.039 (5)	0.032 (5)	0.038 (5)	−0.017 (4)	−0.010 (4)	0.013 (4)
C52	0.052 (5)	0.037 (5)	0.027 (4)	−0.024 (4)	−0.003 (4)	0.006 (3)
C53	0.036 (5)	0.033 (4)	0.023 (4)	−0.013 (4)	−0.006 (3)	0.001 (3)
C54	0.089 (8)	0.040 (5)	0.045 (5)	−0.034 (5)	−0.028 (5)	0.024 (4)
C55	0.050 (5)	0.026 (4)	0.036 (5)	−0.007 (4)	−0.002 (4)	−0.005 (3)
C56	0.053 (5)	0.020 (4)	0.030 (4)	−0.002 (4)	−0.020 (4)	−0.001 (3)
C57	0.024 (4)	0.030 (4)	0.049 (5)	−0.005 (4)	−0.012 (4)	−0.002 (4)
C58	0.023 (4)	0.030 (4)	0.043 (5)	−0.008 (4)	−0.010 (4)	0.004 (3)
C59	0.059 (6)	0.021 (4)	0.041 (5)	−0.001 (4)	−0.016 (4)	0.004 (3)
C60	0.037 (5)	0.029 (5)	0.057 (6)	−0.012 (4)	−0.001 (4)	0.005 (4)
C61	0.042 (5)	0.009 (3)	0.033 (4)	−0.011 (3)	0.001 (4)	0.002 (3)
C62	0.041 (5)	0.033 (5)	0.027 (4)	−0.016 (4)	−0.011 (4)	0.015 (3)
C63	0.039 (5)	0.045 (5)	0.031 (4)	−0.015 (4)	−0.014 (4)	0.003 (4)
C64	0.032 (4)	0.041 (5)	0.022 (4)	−0.015 (4)	−0.007 (3)	0.002 (3)
C65	0.053 (6)	0.043 (5)	0.042 (5)	−0.016 (5)	−0.015 (4)	0.015 (4)
C66	0.046 (5)	0.042 (5)	0.034 (4)	−0.010 (4)	−0.012 (4)	−0.010 (4)

### Geometric parameters (Å, °)

**Table:**

Fe1—N7	2.165 (6)	C16—H16A	0.9600
Fe1—N4	2.184 (6)	C16—H16B	0.9600
Fe1—N5	2.218 (6)	C16—H16C	0.9600
Fe1—N1	2.223 (6)	C17—H17A	0.9700
Fe1—Cl1^i^	2.483 (3)	C17—H17B	0.9700
Fe1—Cl1	2.484 (2)	C18—C19	1.378 (10)
Fe1—Fe1^i^	3.777 (2)	C18—C21	1.499 (10)
Fe2—N11	2.171 (6)	C19—C20	1.386 (10)
Fe2—N15	2.191 (6)	C19—H19A	0.9300
Fe2—N9	2.204 (7)	C20—C22	1.509 (10)
Fe2—N13	2.233 (6)	C21—H21A	0.9600
Fe2—Cl3	2.414 (3)	C21—H21B	0.9600
Fe2—Cl2	2.481 (3)	C21—H21C	0.9600
Fe2—Fe3	3.7028 (17)	C22—H22A	0.9600
Fe3—N19	2.158 (6)	C22—H22B	0.9600
Fe3—N23	2.170 (6)	C22—H22C	0.9600
Fe3—N21	2.202 (6)	C23—C24	1.337 (12)
Fe3—N17	2.213 (6)	C23—C26	1.514 (11)
Fe3—Cl2	2.444 (3)	C24—C25	1.380 (11)
Fe3—Cl3	2.482 (3)	C24—H24A	0.9300
Fe4—Cl6	2.173 (3)	C25—C27	1.498 (11)
Fe4—Cl7	2.187 (3)	C26—H26A	0.9600
Fe4—Cl5	2.188 (3)	C26—H26B	0.9600
Fe4—Cl4	2.218 (3)	C26—H26C	0.9600
Fe5—Cl11	2.163 (3)	C27—H27A	0.9600
Fe5—Cl10	2.177 (3)	C27—H27B	0.9600
Fe5—Cl9	2.186 (3)	C27—H27C	0.9600
Fe5—Cl8	2.210 (2)	C28—H28A	0.9700
Fe6—Cl15	2.174 (3)	C28—H28B	0.9700
Fe6—Cl13	2.180 (3)	C29—C30	1.390 (11)
Fe6—Cl14	2.187 (3)	C29—C32	1.485 (10)
Fe6—Cl12	2.216 (3)	C30—C31	1.406 (10)
Cl1—Fe1^i^	2.483 (3)	C30—H30A	0.9300
N1—C3	1.336 (9)	C31—C33	1.482 (10)
N1—N2	1.376 (8)	C32—H32A	0.9600
N2—C1	1.339 (10)	C32—H32B	0.9600
N2—C6	1.427 (10)	C32—H32C	0.9600
N3—C7	1.344 (9)	C33—H33A	0.9600
N3—N4	1.377 (8)	C33—H33B	0.9600
N3—C6	1.446 (9)	C33—H33C	0.9600
N4—C9	1.349 (9)	C34—C35	1.360 (13)
N5—C14	1.321 (9)	C34—C37	1.507 (12)
N5—N6	1.389 (8)	C35—C36	1.398 (12)
N6—C12	1.352 (9)	C35—H35A	0.9300
N6—C17	1.440 (9)	C36—C38	1.473 (12)
N7—C20	1.350 (9)	C37—H37A	0.9600
N7—N8	1.380 (8)	C37—H37B	0.9600
N8—C18	1.339 (9)	C37—H37C	0.9600
N8—C17	1.448 (9)	C38—H38A	0.9600
N9—C25	1.343 (10)	C38—H38B	0.9600
N9—N10	1.374 (8)	C38—H38C	0.9600
N10—C23	1.358 (10)	C39—H39A	0.9700
N10—C28	1.444 (10)	C39—H39B	0.9700
N11—C31	1.338 (9)	C40—C41	1.362 (11)
N11—N12	1.392 (8)	C40—C43	1.489 (11)
N12—C29	1.346 (9)	C41—C42	1.407 (10)
N12—C28	1.462 (9)	C41—H41A	0.9300
N13—C36	1.325 (10)	C42—C44	1.478 (10)
N13—N14	1.388 (8)	C43—H43A	0.9600
N14—C34	1.338 (10)	C43—H43B	0.9600
N14—C39	1.432 (10)	C43—H43C	0.9600
N15—C42	1.340 (9)	C44—H44A	0.9600
N15—N16	1.373 (8)	C44—H44B	0.9600
N16—C40	1.350 (9)	C44—H44C	0.9600
N16—C39	1.457 (9)	C45—C46	1.346 (13)
N17—C47	1.345 (10)	C45—C48	1.503 (11)
N17—N18	1.364 (8)	C46—C47	1.385 (12)
N18—C45	1.351 (10)	C46—H46A	0.9300
N18—C50	1.451 (11)	C47—C49	1.500 (11)
N19—C53	1.358 (9)	C48—H48A	0.9600
N19—N20	1.363 (8)	C48—H48B	0.9600
N20—C51	1.348 (10)	C48—H48C	0.9600
N20—C50	1.445 (10)	C49—H49A	0.9600
N21—C58	1.342 (9)	C49—H49B	0.9600
N21—N22	1.373 (8)	C49—H49C	0.9600
N22—C56	1.364 (10)	C50—H50A	0.9700
N22—C61	1.461 (9)	C50—H50B	0.9700
N23—C64	1.348 (9)	C51—C52	1.354 (11)
N23—N24	1.381 (8)	C51—C54	1.506 (11)
N24—C62	1.342 (9)	C52—C53	1.391 (10)
N24—C61	1.438 (9)	C52—H52A	0.9300
C1—C2	1.357 (12)	C53—C55	1.483 (10)
C1—C5	1.503 (11)	C54—H54A	0.9600
C2—C3	1.406 (11)	C54—H54B	0.9600
C2—H2A	0.9300	C54—H54C	0.9600
C3—C4	1.493 (10)	C55—H55A	0.9600
C4—H4A	0.9600	C55—H55B	0.9600
C4—H4B	0.9600	C55—H55C	0.9600
C4—H4C	0.9600	C56—C57	1.364 (11)
C5—H5A	0.9600	C56—C59	1.485 (10)
C5—H5B	0.9600	C57—C58	1.376 (10)
C5—H5C	0.9600	C57—H57A	0.9300
C6—H6A	0.9700	C58—C60	1.491 (10)
C6—H6B	0.9700	C59—H59A	0.9600
C7—C8	1.369 (11)	C59—H59B	0.9600
C7—C10	1.493 (10)	C59—H59C	0.9600
C8—C9	1.400 (10)	C60—H60A	0.9600
C8—H8A	0.9300	C60—H60B	0.9600
C9—C11	1.473 (10)	C60—H60C	0.9600
C10—H10A	0.9600	C61—H61A	0.9700
C10—H10B	0.9600	C61—H61B	0.9700
C10—H10C	0.9600	C62—C63	1.364 (11)
C11—H11A	0.9600	C62—C65	1.494 (11)
C11—H11B	0.9600	C63—C64	1.393 (11)
C11—H11C	0.9600	C63—H63A	0.9300
C12—C13	1.364 (11)	C64—C66	1.484 (11)
C12—C15	1.508 (10)	C65—H65A	0.9600
C13—C14	1.404 (10)	C65—H65B	0.9600
C13—H13A	0.9300	C65—H65C	0.9600
C14—C16	1.499 (10)	C66—H66A	0.9600
C15—H15A	0.9600	C66—H66B	0.9600
C15—H15B	0.9600	C66—H66C	0.9600
C15—H15C	0.9600		
			
N7—Fe1—N4	175.9 (2)	C14—C16—H16C	109.5
N7—Fe1—N5	84.3 (2)	H16A—C16—H16C	109.5
N4—Fe1—N5	92.5 (2)	H16B—C16—H16C	109.5
N7—Fe1—N1	93.0 (2)	N6—C17—N8	111.7 (6)
N4—Fe1—N1	84.4 (2)	N6—C17—H17A	109.3
N5—Fe1—N1	90.3 (2)	N8—C17—H17A	109.3
N7—Fe1—Cl1^i^	93.18 (17)	N6—C17—H17B	109.3
N4—Fe1—Cl1^i^	89.69 (17)	N8—C17—H17B	109.3
N5—Fe1—Cl1^i^	95.11 (16)	H17A—C17—H17B	107.9
N1—Fe1—Cl1^i^	172.16 (16)	N8—C18—C19	106.3 (6)
N7—Fe1—Cl1	90.80 (16)	N8—C18—C21	123.6 (7)
N4—Fe1—Cl1	92.58 (16)	C19—C18—C21	130.1 (7)
N5—Fe1—Cl1	173.57 (16)	C18—C19—C20	106.4 (6)
N1—Fe1—Cl1	94.09 (16)	C18—C19—H19A	126.8
Cl1^i^—Fe1—Cl1	81.01 (9)	C20—C19—H19A	126.8
N7—Fe1—Fe1^i^	92.62 (17)	N7—C20—C19	111.1 (6)
N4—Fe1—Fe1^i^	91.49 (17)	N7—C20—C22	121.1 (6)
N5—Fe1—Fe1^i^	135.41 (16)	C19—C20—C22	127.7 (6)
N1—Fe1—Fe1^i^	134.28 (15)	C18—C21—H21A	109.5
Cl1^i^—Fe1—Fe1^i^	40.52 (5)	C18—C21—H21B	109.5
Cl1—Fe1—Fe1^i^	40.49 (6)	H21A—C21—H21B	109.5
N11—Fe2—N15	176.1 (2)	C18—C21—H21C	109.5
N11—Fe2—N9	84.6 (2)	H21A—C21—H21C	109.5
N15—Fe2—N9	93.6 (2)	H21B—C21—H21C	109.5
N11—Fe2—N13	91.8 (2)	C20—C22—H22A	109.5
N15—Fe2—N13	84.7 (2)	C20—C22—H22B	109.5
N9—Fe2—N13	89.8 (2)	H22A—C22—H22B	109.5
N11—Fe2—Cl3	89.59 (18)	C20—C22—H22C	109.5
N15—Fe2—Cl3	92.54 (17)	H22A—C22—H22C	109.5
N9—Fe2—Cl3	172.30 (17)	H22B—C22—H22C	109.5
N13—Fe2—Cl3	95.43 (18)	C24—C23—N10	106.9 (7)
N11—Fe2—Cl2	94.50 (17)	C24—C23—C26	131.4 (9)
N15—Fe2—Cl2	89.04 (16)	N10—C23—C26	121.7 (8)
N9—Fe2—Cl2	93.04 (17)	C23—C24—C25	107.5 (8)
N13—Fe2—Cl2	173.29 (17)	C23—C24—H24A	126.3
Cl3—Fe2—Cl2	82.42 (9)	C25—C24—H24A	126.3
N11—Fe2—Fe3	93.45 (17)	N9—C25—C24	110.4 (8)
N15—Fe2—Fe3	90.32 (17)	N9—C25—C27	121.4 (7)
N9—Fe2—Fe3	133.71 (16)	C24—C25—C27	128.1 (8)
N13—Fe2—Fe3	136.52 (17)	C23—C26—H26A	109.5
Cl3—Fe2—Fe3	41.56 (6)	C23—C26—H26B	109.5
Cl2—Fe2—Fe3	40.88 (6)	H26A—C26—H26B	109.5
N19—Fe3—N23	175.4 (2)	C23—C26—H26C	109.5
N19—Fe3—N21	92.1 (2)	H26A—C26—H26C	109.5
N23—Fe3—N21	83.9 (2)	H26B—C26—H26C	109.5
N19—Fe3—N17	84.4 (2)	C25—C27—H27A	109.5
N23—Fe3—N17	93.5 (2)	C25—C27—H27B	109.5
N21—Fe3—N17	91.8 (2)	H27A—C27—H27B	109.5
N19—Fe3—Cl2	92.93 (19)	C25—C27—H27C	109.5
N23—Fe3—Cl2	91.30 (17)	H27A—C27—H27C	109.5
N21—Fe3—Cl2	172.22 (17)	H27B—C27—H27C	109.5
N17—Fe3—Cl2	94.60 (18)	N10—C28—N12	111.5 (6)
N19—Fe3—Cl3	88.04 (18)	N10—C28—H28A	109.3
N23—Fe3—Cl3	94.39 (17)	N12—C28—H28A	109.3
N21—Fe3—Cl3	92.45 (17)	N10—C28—H28B	109.3
N17—Fe3—Cl3	171.44 (18)	N12—C28—H28B	109.3
Cl2—Fe3—Cl3	81.80 (9)	H28A—C28—H28B	108.0
N19—Fe3—Fe2	91.30 (19)	N12—C29—C30	105.6 (6)
N23—Fe3—Fe2	93.09 (17)	N12—C29—C32	124.5 (7)
N21—Fe3—Fe2	132.33 (16)	C30—C29—C32	129.9 (7)
N17—Fe3—Fe2	135.84 (17)	C29—C30—C31	106.8 (7)
Cl2—Fe3—Fe2	41.62 (6)	C29—C30—H30A	126.6
Cl3—Fe3—Fe2	40.19 (6)	C31—C30—H30A	126.6
Cl6—Fe4—Cl7	108.90 (13)	N11—C31—C30	110.4 (6)
Cl6—Fe4—Cl5	110.04 (13)	N11—C31—C33	122.6 (6)
Cl7—Fe4—Cl5	109.23 (11)	C30—C31—C33	127.0 (7)
Cl6—Fe4—Cl4	109.57 (12)	C29—C32—H32A	109.5
Cl7—Fe4—Cl4	108.78 (12)	C29—C32—H32B	109.5
Cl5—Fe4—Cl4	110.28 (12)	H32A—C32—H32B	109.5
Cl11—Fe5—Cl10	110.33 (14)	C29—C32—H32C	109.5
Cl11—Fe5—Cl9	108.39 (13)	H32A—C32—H32C	109.5
Cl10—Fe5—Cl9	111.11 (13)	H32B—C32—H32C	109.5
Cl11—Fe5—Cl8	108.99 (11)	C31—C33—H33A	109.5
Cl10—Fe5—Cl8	108.23 (13)	C31—C33—H33B	109.5
Cl9—Fe5—Cl8	109.77 (10)	H33A—C33—H33B	109.5
Cl15—Fe6—Cl13	109.96 (11)	C31—C33—H33C	109.5
Cl15—Fe6—Cl14	110.51 (12)	H33A—C33—H33C	109.5
Cl13—Fe6—Cl14	109.46 (11)	H33B—C33—H33C	109.5
Cl15—Fe6—Cl12	107.95 (11)	N14—C34—C35	105.5 (7)
Cl13—Fe6—Cl12	109.50 (11)	N14—C34—C37	121.9 (9)
Cl14—Fe6—Cl12	109.43 (11)	C35—C34—C37	132.5 (9)
Fe1^i^—Cl1—Fe1	98.99 (9)	C34—C35—C36	108.0 (8)
Fe3—Cl2—Fe2	97.50 (9)	C34—C35—H35A	126.0
Fe2—Cl3—Fe3	98.25 (10)	C36—C35—H35A	126.0
C3—N1—N2	105.4 (6)	N13—C36—C35	109.5 (8)
C3—N1—Fe1	135.0 (5)	N13—C36—C38	122.8 (8)
N2—N1—Fe1	118.3 (4)	C35—C36—C38	127.6 (8)
C1—N2—N1	111.1 (6)	C34—C37—H37A	109.5
C1—N2—C6	129.3 (7)	C34—C37—H37B	109.5
N1—N2—C6	119.5 (6)	H37A—C37—H37B	109.5
C7—N3—N4	111.8 (6)	C34—C37—H37C	109.5
C7—N3—C6	129.6 (6)	H37A—C37—H37C	109.5
N4—N3—C6	118.5 (5)	H37B—C37—H37C	109.5
C9—N4—N3	104.8 (5)	C36—C38—H38A	109.5
C9—N4—Fe1	134.9 (5)	C36—C38—H38B	109.5
N3—N4—Fe1	120.2 (4)	H38A—C38—H38B	109.5
C14—N5—N6	104.7 (6)	C36—C38—H38C	109.5
C14—N5—Fe1	137.0 (5)	H38A—C38—H38C	109.5
N6—N5—Fe1	117.7 (4)	H38B—C38—H38C	109.5
C12—N6—N5	111.1 (6)	N14—C39—N16	112.4 (6)
C12—N6—C17	128.5 (6)	N14—C39—H39A	109.1
N5—N6—C17	120.4 (6)	N16—C39—H39A	109.1
C20—N7—N8	103.5 (5)	N14—C39—H39B	109.1
C20—N7—Fe1	135.9 (5)	N16—C39—H39B	109.1
N8—N7—Fe1	120.6 (4)	H39A—C39—H39B	107.9
C18—N8—N7	112.6 (6)	N16—C40—C41	106.0 (7)
C18—N8—C17	128.4 (6)	N16—C40—C43	124.3 (7)
N7—N8—C17	119.0 (5)	C41—C40—C43	129.7 (7)
C25—N9—N10	104.4 (6)	C40—C41—C42	106.8 (6)
C25—N9—Fe2	135.2 (5)	C40—C41—H41A	126.6
N10—N9—Fe2	118.0 (5)	C42—C41—H41A	126.6
C23—N10—N9	110.8 (6)	N15—C42—C41	110.3 (6)
C23—N10—C28	129.7 (7)	N15—C42—C44	122.0 (6)
N9—N10—C28	119.3 (6)	C41—C42—C44	127.7 (7)
C31—N11—N12	104.5 (5)	C40—C43—H43A	109.5
C31—N11—Fe2	135.4 (5)	C40—C43—H43B	109.5
N12—N11—Fe2	120.0 (4)	H43A—C43—H43B	109.5
C29—N12—N11	112.6 (6)	C40—C43—H43C	109.5
C29—N12—C28	129.1 (6)	H43A—C43—H43C	109.5
N11—N12—C28	118.2 (5)	H43B—C43—H43C	109.5
C36—N13—N14	104.9 (6)	C42—C44—H44A	109.5
C36—N13—Fe2	133.4 (5)	C42—C44—H44B	109.5
N14—N13—Fe2	115.9 (5)	H44A—C44—H44B	109.5
C34—N14—N13	112.1 (7)	C42—C44—H44C	109.5
C34—N14—C39	129.0 (7)	H44A—C44—H44C	109.5
N13—N14—C39	118.9 (6)	H44B—C44—H44C	109.5
C42—N15—N16	104.3 (5)	C46—C45—N18	105.8 (7)
C42—N15—Fe2	135.5 (5)	C46—C45—C48	130.8 (9)
N16—N15—Fe2	120.0 (4)	N18—C45—C48	123.3 (9)
C40—N16—N15	112.6 (6)	C45—C46—C47	108.1 (8)
C40—N16—C39	129.4 (6)	C45—C46—H46A	125.9
N15—N16—C39	117.6 (5)	C47—C46—H46A	125.9
C47—N17—N18	104.8 (6)	N17—C47—C46	109.3 (8)
C47—N17—Fe3	136.7 (5)	N17—C47—C49	121.3 (7)
N18—N17—Fe3	118.5 (5)	C46—C47—C49	129.3 (9)
C45—N18—N17	111.9 (7)	C45—C48—H48A	109.5
C45—N18—C50	128.5 (7)	C45—C48—H48B	109.5
N17—N18—C50	119.6 (6)	H48A—C48—H48B	109.5
C53—N19—N20	105.1 (6)	C45—C48—H48C	109.5
C53—N19—Fe3	134.9 (5)	H48A—C48—H48C	109.5
N20—N19—Fe3	119.6 (4)	H48B—C48—H48C	109.5
C51—N20—N19	111.5 (6)	C47—C49—H49A	109.5
C51—N20—C50	128.7 (7)	C47—C49—H49B	109.5
N19—N20—C50	119.8 (6)	H49A—C49—H49B	109.5
C58—N21—N22	104.3 (6)	C47—C49—H49C	109.5
C58—N21—Fe3	136.6 (5)	H49A—C49—H49C	109.5
N22—N21—Fe3	117.6 (5)	H49B—C49—H49C	109.5
C56—N22—N21	111.7 (6)	N20—C50—N18	112.3 (7)
C56—N22—C61	128.5 (6)	N20—C50—H50A	109.1
N21—N22—C61	119.8 (6)	N18—C50—H50A	109.1
C64—N23—N24	105.1 (6)	N20—C50—H50B	109.1
C64—N23—Fe3	134.8 (5)	N18—C50—H50B	109.1
N24—N23—Fe3	119.1 (4)	H50A—C50—H50B	107.9
C62—N24—N23	111.3 (6)	N20—C51—C52	106.8 (7)
C62—N24—C61	129.7 (6)	N20—C51—C54	123.1 (7)
N23—N24—C61	118.4 (6)	C52—C51—C54	130.1 (8)
N2—C1—C2	107.5 (7)	C51—C52—C53	107.4 (7)
N2—C1—C5	122.4 (8)	C51—C52—H52A	126.3
C2—C1—C5	130.0 (8)	C53—C52—H52A	126.3
C1—C2—C3	106.4 (7)	N19—C53—C52	109.1 (7)
C1—C2—H2A	126.8	N19—C53—C55	122.9 (6)
C3—C2—H2A	126.8	C52—C53—C55	128.0 (7)
N1—C3—C2	109.7 (7)	C51—C54—H54A	109.5
N1—C3—C4	123.6 (6)	C51—C54—H54B	109.5
C2—C3—C4	126.7 (7)	H54A—C54—H54B	109.5
C3—C4—H4A	109.5	C51—C54—H54C	109.5
C3—C4—H4B	109.5	H54A—C54—H54C	109.5
H4A—C4—H4B	109.5	H54B—C54—H54C	109.5
C3—C4—H4C	109.5	C53—C55—H55A	109.5
H4A—C4—H4C	109.5	C53—C55—H55B	109.5
H4B—C4—H4C	109.5	H55A—C55—H55B	109.5
C1—C5—H5A	109.5	C53—C55—H55C	109.5
C1—C5—H5B	109.5	H55A—C55—H55C	109.5
H5A—C5—H5B	109.5	H55B—C55—H55C	109.5
C1—C5—H5C	109.5	C57—C56—N22	105.5 (6)
H5A—C5—H5C	109.5	C57—C56—C59	132.1 (8)
H5B—C5—H5C	109.5	N22—C56—C59	122.3 (8)
N2—C6—N3	113.0 (6)	C56—C57—C58	107.6 (7)
N2—C6—H6A	109.0	C56—C57—H57A	126.2
N3—C6—H6A	109.0	C58—C57—H57A	126.2
N2—C6—H6B	109.0	N21—C58—C57	110.8 (7)
N3—C6—H6B	109.0	N21—C58—C60	121.6 (7)
H6A—C6—H6B	107.8	C57—C58—C60	127.6 (7)
N3—C7—C8	106.6 (6)	C56—C59—H59A	109.5
N3—C7—C10	124.3 (7)	C56—C59—H59B	109.5
C8—C7—C10	129.1 (7)	H59A—C59—H59B	109.5
C7—C8—C9	106.9 (7)	C56—C59—H59C	109.5
C7—C8—H8A	126.6	H59A—C59—H59C	109.5
C9—C8—H8A	126.6	H59B—C59—H59C	109.5
N4—C9—C8	109.7 (7)	C58—C60—H60A	109.5
N4—C9—C11	122.4 (7)	C58—C60—H60B	109.5
C8—C9—C11	127.9 (7)	H60A—C60—H60B	109.5
C7—C10—H10A	109.5	C58—C60—H60C	109.5
C7—C10—H10B	109.5	H60A—C60—H60C	109.5
H10A—C10—H10B	109.5	H60B—C60—H60C	109.5
C7—C10—H10C	109.5	N24—C61—N22	112.5 (6)
H10A—C10—H10C	109.5	N24—C61—H61A	109.1
H10B—C10—H10C	109.5	N22—C61—H61A	109.1
C9—C11—H11A	109.5	N24—C61—H61B	109.1
C9—C11—H11B	109.5	N22—C61—H61B	109.1
H11A—C11—H11B	109.5	H61A—C61—H61B	107.8
C9—C11—H11C	109.5	N24—C62—C63	106.8 (7)
H11A—C11—H11C	109.5	N24—C62—C65	122.7 (7)
H11B—C11—H11C	109.5	C63—C62—C65	130.5 (7)
N6—C12—C13	107.0 (6)	C62—C63—C64	107.3 (7)
N6—C12—C15	123.5 (7)	C62—C63—H63A	126.3
C13—C12—C15	129.5 (7)	C64—C63—H63A	126.3
C12—C13—C14	106.0 (7)	N23—C64—C63	109.4 (7)
C12—C13—H13A	127.0	N23—C64—C66	122.8 (7)
C14—C13—H13A	127.0	C63—C64—C66	127.8 (7)
N5—C14—C13	111.3 (6)	C62—C65—H65A	109.5
N5—C14—C16	121.6 (7)	C62—C65—H65B	109.5
C13—C14—C16	127.1 (7)	H65A—C65—H65B	109.5
C12—C15—H15A	109.5	C62—C65—H65C	109.5
C12—C15—H15B	109.5	H65A—C65—H65C	109.5
H15A—C15—H15B	109.5	H65B—C65—H65C	109.5
C12—C15—H15C	109.5	C64—C66—H66A	109.5
H15A—C15—H15C	109.5	C64—C66—H66B	109.5
H15B—C15—H15C	109.5	H66A—C66—H66B	109.5
C14—C16—H16A	109.5	C64—C66—H66C	109.5
C14—C16—H16B	109.5	H66A—C66—H66C	109.5
H16A—C16—H16B	109.5	H66B—C66—H66C	109.5

Symmetry codes: (i) −*x*+1, −*y*+1, −*z*+1.
